# Extracorporeal shock wave treatment attenuated left ventricular dysfunction and remodeling in mini-pig with cardiorenal syndrome

**DOI:** 10.18632/oncotarget.18287

**Published:** 2017-05-30

**Authors:** Jiunn-Jye Sheu, Hani E.E. Ali, Ben-Chung Cheng, Hsin-Ju Chiang, Pei-Hsun Sung, Kuan-Hung Chen, Chih-Chao Yang, Yen-Ta Chen, John Y. Chiang, Pao-Yuan Lin, Sarah Chua, Han-Tan Chai, Sheng-Ying Chung, Cheuk-Kwan Sun, Hon-Kan Yip

**Affiliations:** ^1^ Division of Thoracic and Cardiovascular Surgery, Department of Surgery, Kaohsiung Chang Gung Memorial Hospital and Chang Gung University College of Medicine, Kaohsiung, Taiwan; ^2^ Division of Nephrology, Department of Internal Medicine, Kaohsiung Chang Gung Memorial Hospital and Chang Gung University College of Medicine, Kaohsiung, Taiwan; ^3^ Department of Obstetrics and Gynecology, Kaohsiung Chang Gung Memorial Hospital and Chang Gung University College of Medicine, Kaohsiung, Taiwan; ^4^ Division of Cardiology, Department of Internal Medicine, Kaohsiung Chang Gung Memorial Hospital and Chang Gung University College of Medicine, Kaohsiung, Taiwan; ^5^ Department of Anesthesiology, Kaohsiung Chang Gung Memorial Hospital and Chang Gung University College of Medicine, Kaohsiung, Taiwan; ^6^ Division of Urology, Department of Surgery, Kaohsiung Chang Gung Memorial Hospital and Chang Gung University College of Medicine, Kaohsiung, Taiwan; ^7^ Department of Computer Science and Engineering, National Sun Yat-Sen University, Kaohsiung, Taiwan; ^8^ Department of Plastic and Reconstructive Surgery, Kaohsiung Chang Gung Memorial Hospital and Chang Gung University College of Medicine, Kaohsiung, Taiwan; ^9^ Department of Emergency Medicine, E-Da Hospital, I-Shou University School of Medicine for International Students, Kaohsiung, Taiwan; ^10^ Institute for Translational Research in Biomedicine, Kaohsiung Chang Gung Memorial Hospital and Chang Gung University College of Medicine, Kaohsiung, Taiwan; ^11^ Center for Shockwave Medicine and Tissue Engineering, Kaohsiung Chang Gung Memorial Hospital and Chang Gung University College of Medicine, Kaohsiung, Taiwan; ^12^ Department of Medical Research, China Medical University Hospital, China Medical University, Taichung, Taiwan; ^13^ Department of Nursing, Asia University, Taichung, Taiwan

**Keywords:** myocardial ischemia, LV remodeling, cardiorenal syndrome, angiogenesis, extracorporeal shock wave

## Abstract

This study tested the hypothesis that extracorporeal shock wave (ECSW) treatment can improve ischemia-induced left ventricular (LV) dysfunction in mini-pig with co-existing chronic kidney disease (CKD). LV ischemia in mini-pigs was induced by applying an ameroid constrictor over mid-left anterior descending artery (LAD), while model of CKD was established by right nephrectomy with partial ligation of left renal arterioles 2 weeks before LAD constriction. Thirty mini-pigs were randomly divided into group 1 (sham-control), group 2 (LV-ischemia), group 3 (LV-ischemia + CKD), Group 4 [LV-ischemia + ECSW (applied 1200 shots at 0.1 mJ/m^2^/equally to 4-ischemic regions by day-90 after LAD constriction], and group 5 (LV-ischemia-CKD + ECSW). By day-180 after CKD induction, echocardiography showed that LV ejection fraction (LVEF) was highest in group 1, lowest in group 3, significantly lower in group 2 than that in groups 4 and 5, and significantly lower in group 5 than that in group 4, whereas LV-end systolic and diastolic dimensions displayed an opposite pattern (all p<0.001). Protein expressions of oxidative-stress (NOX-1/NOX-2/oxidized protein), apoptotic (cleaved-caspase-3/cleaved-PARP/mitochondrial-Bax), fibrotic (TGF-β/Smad3), pressure/volume-overload (BNP/β-MHC), endothelial (CD31/vWF) and mitochondrial-integrity (PGC-1/mitochondrial-cytochrome-C) biomarkers exhibited a pattern identical to that of LVEF, whereas angiogenesis factors (VEGF/CXCR4/SDF-1α) showed significant progressive increase among all groups (all p<0.0001). Microscopic findings of CD31+cells/vWF+cells/small-vessel density/sarcomere-length showed an identical pattern, whereas collagen-deposition area/fibrotic area/apoptotic nuclei expressed an opposite pattern compared to that of LVEF among all groups (all p<0.0001). In conclusion, CKD aggravated ischemia-induced LV dysfunction and remodeling and molecular-cellular perturbations that were reversed by ECSW treatment.

## INTRODUCTION

Not only is optimal pharmacological therapy of limited help for patients with diffuse obstructive coronary artery disease (CAD) [1, 2], but interventional therapies via either percutaneous coronary intervention (PCI) or coronary artery bypass surgery (CABG) are also unsuitable for this patient population [3, 4]. To date, an effective treatment for patients with diffuse obstructive CAD who are not candidates for catheter-based or surgical intervention is still pending.

There is universal consensus on that chronic kidney disease (CKD) [i.e., in advanced stage or end-stage renal disease (ESRD)] is one of the most crucial contributors to the development of diffuse obstructive CAD, leading to cardiorenal disease. In fact, abundant data have shown that cardiac and renal diseases remain the two major health care burdens worldwide [5–9]. Not only are the essential etiologies similar between the two diseases (i.e., endothelial dysfunction and arteriosclerosis, diabetes mellitus, hypertension), but the treatment strategies are also comparable [10–12]. These two often co-existing diseases are both common and increasingly encountered with perpetuating effects on each other (i.e., cardiorenal syndrome, CRS) [10–14].

Growing data have shown that enhancing angiogenesis is one of the effective management strategies for retorting blood flow to ischemic area [2, 15–20], resulting in alleviation of ischemia-related organ dysfunction, especially in patients with diffuse CAD unsuitable for interventional therapies [2, 17, 18]. Extracorporeal shock wave (ECSW) is a longitudinal acoustic wave traveling with the speed of ultrasound in water through body tissue. In addition to the mechanical action originally developed for the treatment of lithotripsy, it is now widely used clinically because of its well-documented pleotropic effects [15, 19–24]. It generates a single pressure pulse with a short needle-like positive spike < 1 μs in duration and up to 100 MPa in amplitude, followed by a tensile part of several microseconds with lower amplitude that is known to exert the “cavitation effect” [25]. A number of experimental studies have shown that ECSW therapy effectively improves ischemia-related organ dysfunction [15, 19, 20, 26] mainly through increased angiogenesis/vasculogenesis, thereby restoring blood flow in ischemic region [15, 19, 20, 27–29]. Interestingly, an increasing number of clinical studies has shown significant alleviation of symptoms in patients with refractory angina [30–33] and ischemia-related left ventricular (LV) dysfunction following ECSW treatment [31, 33]. However, whether ECSW therapy can improve CRS-related angina and LV dysfunction is currently unclear. This issue raises the need for experimental studies to investigate the therapeutic effect of ECSW against myocardial ischemia and LV dysfunction in the setting of CRS prior to its potential clinical application.

## RESULTS

### Time courses of circulating levels of creatinine, flow cytometric analysis of circulating level of endothelial progenitor cells (EPCs), and kidney injury score by day 180 after CKD induction (Figure 1)

As expected, the circulating creatinine level at baseline did not differ among the five groups. However, the circulating creatinine level at days 30, 90 and 180 after CKD significantly higher in group 3 (i.e., LVIs-CKD) than in other groups, and significantly higher in group 5 (i.e., LVIs-CKD-ECSW) than that in group 1 (i.e., SC), groups 2 (LVIs) and 4 (LVIs-ECSW), but it showed no difference among the groups 1, 2 and 4.

**Figure 1:**
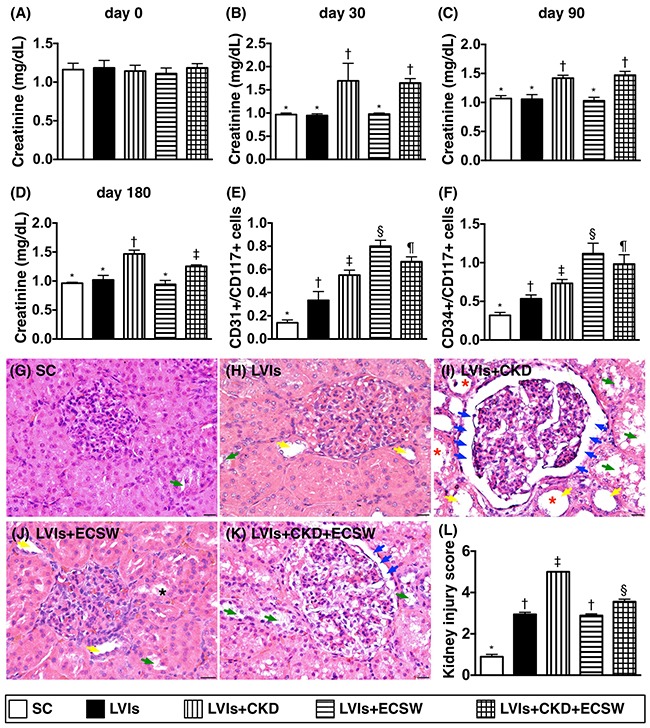
Serial changes of circulating creatinine levels, flow cytometric analysis of circulating level of endothelial progenitor cells (EPCs) and kidney injury score by day 180 after CKD induction **(A)** Circulating creatinine level at baseline, p>0.5. **(B)** Circulating creatinine level at day 30, * vs. ‡, p<0.0001. **(C)** Circulating creatinine level at day 90, * vs. ‡, p<0.001. **(D)** Circulating creatinine level at day 180, * vs. other groups with different symbols (†, ‡), p<0.0001. **(E)** Number of circulating CD31^+^/CD117^+^ cells at day 180, * vs. other groups with different symbols (†, ‡, §, ¶), p<0.0001. **(F)** Number of circulating CD34^+^/CD117^+^ cells at day 180, * vs. other groups with different symbols (†, ‡, §, ¶), p<0.0001. **(G to K)** Light microscopic findings of H&E stain (400x) demonstrating significantly higher degree of loss of brush border in renal tubules (yellow arrows), tubular necrosis (green arrows), tubular dilatation (red asterisk) protein cast formation (black asterisk), and dilatation of Bowman's capsule (blue arrows) in LVIs-CKD group than in other groups. **(L)** * vs. other groups with different symbols (†, ‡, §), p<0.0001. Scale bars in right lower corner represent 50μm. All statistical analyses were performed by one-way ANOVA, followed by Bonferroni multiple comparison post hoc test (n=6 for each group). Symbols (*, †, ‡, §, ¶) indicate significance (at 0.05 level). SC: sham-operative control; LVIs: left ventricular ischemia; CKD: chronic kidney disease; ECSW: extracorporeal shock wave.

By the end of the study period (i.e., at day 180), the circulating levels of EPCs (i.e., CD31^+^CD117^+^, CD34^+^CD117^+^) were lowest in group 1 and highest in group 4, significantly higher in group 5 than those in groups 2 and 3, and significantly higher in group 3 than those in group 2, suggesting that: 1) an increase in circulating EPCs was an intrinsic response to ischemic stimulation that was suppressed by CKD; 2) ECSW treatment enhanced circulating level of EPCs.

Light microscopy of hematoxylin and eosin (H & E)-stained kidney sections revealed that kidney injury score was lowest in group 1 and highest in group 3, significantly higher in group 5 than that in groups 2 and 4, but it showed no difference in the latter two groups.

### Time courses of transthoracic echocardiographic examination (Table 1)

Table 1 shows serial echocardiographic findings prior to and at days 44, 90, and 180 after CKD induction. The baseline parameters of transthoracic echocardiography including left ventricular end-diastolic diameter (LVEDd), left ventricular end-systolic diameter (LVESd), and left ventricular ejection fraction (LVEF) did not differ among the five groups. However, by day 44 after CKD induction (i.e., at day 30 after myocardial ischemia induction by application of ameroid constrictor to left anterior descending artery [LAD]), the LVEF was significantly lower, whereas the LVEDd and LVESd were significantly higher in groups 2 to 5 than that in group 1, but these parameters did not differ among the groups 2 to 5. Consistently, by day 90 after CKD induction, these three parameters showed an identical pattern of day 44 among the five groups. Moreover, by day 180 (i.e., at the end of study period), LVEF was highest in group 1 and lowest in group 3, significantly lower in group 2 than that in group 4 and 5, and significantly lower in group 5 than that in group 4. On the other hand, LVEDd and LVESd showed an opposite pattern compared to that of LVEF among the five groups.

**Table 1:** Time courses of transthoracic echocardiographic findings among five groups

Variables	Group 1 (n=6)	Group 2 (n=6)	Group 3 (n=6)	Group 4 (n=6)	Group 5 (n=6)	p-value
Day 0						
LVEDd (mm)	34.91 ± 6.81	32.36 ± 5.0	34.0 ± 3.85	33.55 ± 7.0	35.90 ± 3.72	0.712
LVESd (mm)	21.95 ± 4.92	20.76 ± 3.89	21.59 ± 1.70	20.68 ± 5.10	23.11 ± 2.87	0.531
LVEF (%)	68.58 ± 3.67	67.04 ± 3.76	66.92 ± 3.69	67.39 ± 4.15	66.18 ± 2.96	0.882
Day 44*						
LVEDd (mm)	34.41 ± 4.82^a^	39.22 ± 2.31^b^	39.96 ± 3.43^b^	40.14 ± 3.24^b^	38.18 ± 3.12^b^	<0.001
LVESd (mm)	21.15 ± 2.92^a^	28.09 ± 1.61^b^	29.01 ± 1.62^b^	28.98 ± 2.08^b^	28.31 ± 1.96^b^	<0.001
LVEF (%)	67.86 ± 3.67^a^	55.36 ± 1.88^b^	55.26 ± 4.76^b^	57.84 ± 5.98^b^	57.05 ± 2.80^b^	<0.001
Day 90†						
LVEDd (mm)	35.56 ± 2.17^a^	41.01 ± 5.18^b^	40.78 ± 6.61^b^	41.85 ± 3.46^b^	38.92 ± 5.10^b^	<0.01
LVESd (mm)	22.70 ± 1.89^a^	30.82 ± 3.44^b^	30.10 ± 4.43^b^	31.39 ± 2.37^b^	28.18 ± 3.50^b^	<0.001
LVEF (%)	66.82 ± 3.29^a^	50.27 ± 2.15^b^	51.47 ± 4.13^b^	50.77 ± 4.35^b^	51.96 ± 4.09^b^	<0.001
Day 180						
LVEDd (mm)	36.26 ± 2.47^a^	45.59 ± 2.62^b^	45.43 ± 4.97^b^	45.05 ± 2.76^b^	46.23 ± 3.41^b^	<0.0001
LVESd (mm)	23.21 ± 2.02^a^	35.19 ± 4.43^b^	36.38 ± 4.46^b^	31.46 ± 1.61^c^	32.17 ± 1.45^c^	<0.0001
LVEF (%)	66.27 ± 3.72^a^	45.03 ± 9.59^b^	40.60 ± 5.05^c^	59.61 ± 3.93^d^	56.41 ± 2.79^e^	<0.0001

Data are expressed as mean ± SD or (%).

Group 1 = sham-operated control; group 2 = left ventricular ischemia (LVIs) by ameroid constrictor applied to left anterior descending artery; group 3 = LVIs + chronic kidney disease (CKD) by right nephrectomy and half (1/2) of the left renal artery ligation (i.e., LVIs + CKD); group 4 = LVIs + extracorporeal shock wave (ECSW); group 5 = LVIs + CKD + ECSW.

* indicated the timing was 44 days after CKD induction.

† indicated the ECSW was applied to groups 4 and 5 by day 90 after CKD induction.

LVEDd: left ventricular end-diastolic dimension; LVESd: left ventricular end-systolic dimension; LVEF: left ventricular ejection fraction.

Letters (^a, b, c, d, e^) indicate significant difference (at 0.05 level) by Tukey multiple comparison procedure.

### Fibrosis and collagen-deposition area in left ventricular (LV) myocardium by day 180 after CKD induction (Figure 2)

Microscopically, Masson's trichrome staining demonstrated that the fibrotic area of LV myocardium was lowest in group 1 and highest in group 3, significantly higher in group 2 than that in groups 4 and 5, and significantly higher in group 5 than that in group 4. In addition, immunohistochemical (IHC) staining of collagen deposition area, another myocardial fibrosis indicator, exhibited a pattern identical to that of Masson's trichrome staining among the five groups.

**Figure 2:**
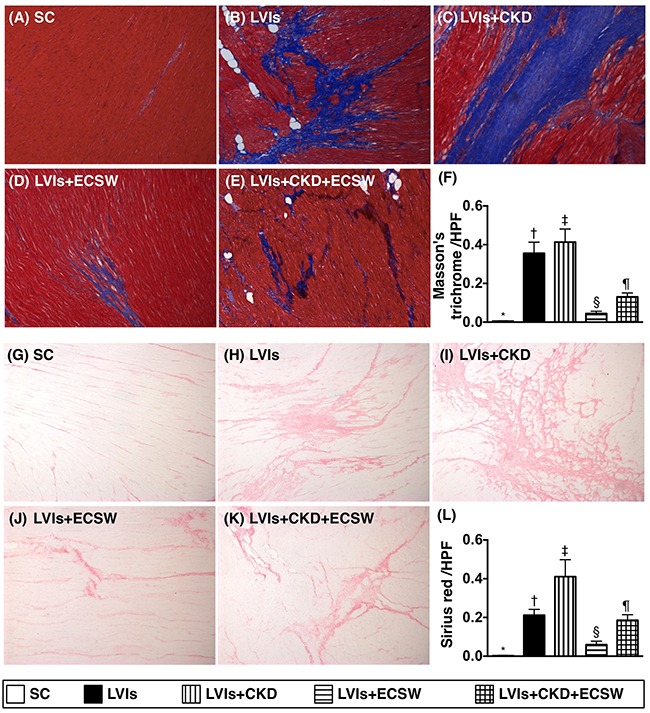
Expressions of fibrosis and collagen deposition in LV myocardium by day 180 after CKD induction **(A to E)** Immunohistochemical (IHC) microscopic finding (100x) of Masson's trichrome for identification of fibrotic area (blue color) in LV myocardium. **(F)** Analytical result of fibrotic area, * vs. other groups with different symbols (†, ‡, §, ¶), p<0.0001. **(G to K)** IHC microscopic finding (100x) of Sirius red for identification of collagen-deposition area (pink color) in LV myocardium. **(L)** Analytical result of collagen-deposition area, * vs. other groups with different symbols (†, ‡, §, ¶), p<0.0001. Scale bars in right lower corner represent 100μm. All statistical analyses were performed by one-way ANOVA, followed by Bonferroni multiple comparison post hoc test (n=6 for each group). Symbols (*, †, ‡, §, ¶) indicate significance (at 0.05 level). SC: sham-operative control; LVIs: left ventricular ischemia; CKD: chronic kidney disease; ECSW: extracorporeal shock wave.

### Small vessel density and apoptotic nuclei in LV myocardium by day 180 after CKD induction (Figure 3)

Alpha- smooth muscle actin (SMA) staining demonstrated that the number of small vessels (i.e., ≤25.0μm in diameter) was highest in group 1 and lowest in group 3, significantly lower in group 2 than that in groups 4 and 5, and significantly lower in group 5 than in group 4. Besides, terminal deoxynucleotidyl transferase dUTP nick end labeling (TUNEL) assay revealed that the number of apoptotic nuclei, an indicator of cellular apoptosis, exhibited an opposite pattern compared to that of the number of small vessels among the five groups.

**Figure 3:**
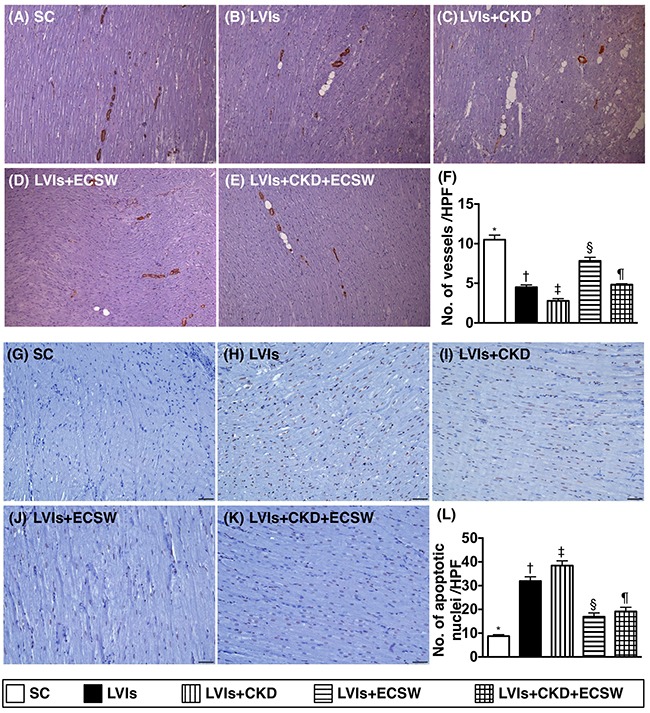
Expressions of small vessels and apoptotic cells in LV myocardium by day 180 after CKD induction **(A to E)** Microscopic finding (100x) of α-SMA stain for identification of small vessels (gray color) in LV myocardium. **(F)** Analytic results of number of small vessel (≤ 25μm), * vs. other groups with different symbols (†, ‡, §, ¶), p<0.0001. Scale bars in right lower corner represent 100μm. **(G to K)** Microscopic finding (200x) of TUNEL assay for identification of apoptotic nuclei (gray color) in LV myocardium. **(L)** Analytical result of number of apoptotic nuclei, * vs. other groups with different symbols (†, ‡, §, ¶), p<0.0001. Scale bars in right lower corner represent 50μm. All statistical analyses were performed by one-way ANOVA, followed by Bonferroni multiple comparison post hoc test (n=6 for each group). Symbols (*, †, ‡, §, ¶) indicate significance (at 0.05 level). SC: sham-operative control; LVIs: left ventricular ischemia; CKD: chronic kidney disease; ECSW: extracorporeal shock wave.

### Cellular angiogenesis in LV myocardium by day 180 after CKD induction (Figure 4)

Immunofluorescence (IF) staining showed that the numbers of cells positive for CD31 and von Willebrand factor (vWF), two endothelial cells markers, were lowest in group 3 and highest in group 1, significantly lower in group 2 than those in groups 4 and 5, and significantly lower in group 5 than those in group 4.

**Figure 4:**
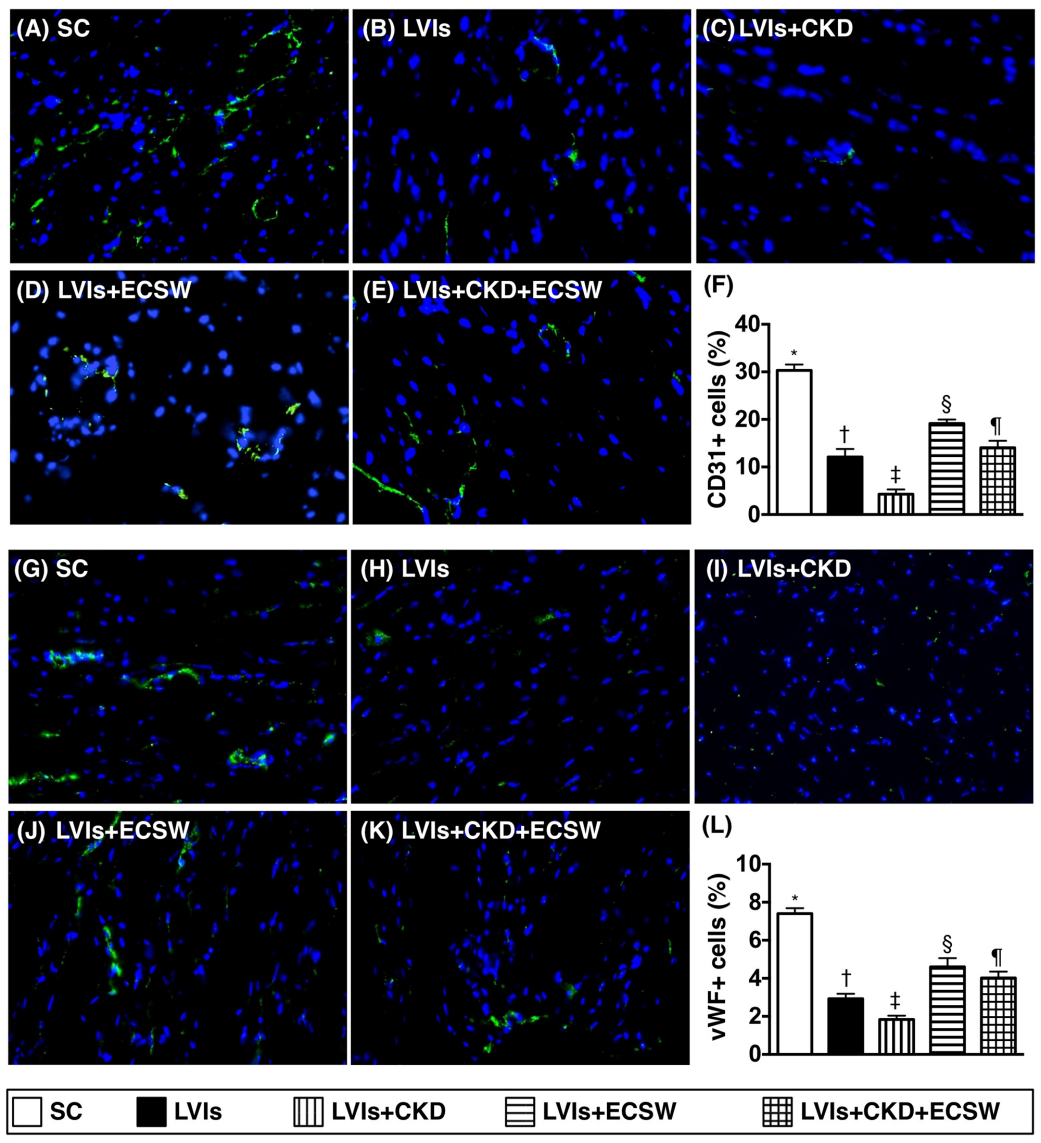
Cellular level of angiogenesis in LV myocardium by day 180 after CKD induction **(A to E)** Immunofluorescent (IF) microscopic finding (400x) for identifying the expression of CD31+ cells (green color). **(F)** Analytical result of number of CD31+ cells, * vs. other groups with different symbols (†, ‡, §, ¶), p<0.0001. **(G to K)** IF microscopic finding (400x) for identifying the expression of von Willebrand factor (vWF)+ cells (green color). **(L)** Analytical result of number of vWF+ cells, * vs. other groups with different symbols (†, ‡, §, ¶), p<0.0001. Scale bars in right lower corner represent 20μm. All statistical analyses were performed by one-way ANOVA, followed by Bonferroni multiple comparison post hoc test (n=6 for each group). Symbols (*, †, ‡, §, ¶) indicate significance (at 0.05 level). SC: sham-operative control; LVIs: left ventricular ischemia; CKD: chronic kidney disease; ECSW: extracorporeal shock wave.

### Protein expressions of angiogenesis factors in LV myocardium by day 180 after CKD induction (Figure 5)

The protein expressions of CD31 and vWF, two indicators of angiogenesis, were lowest in group 3 and highest in group 1, significantly lower in group 2 than those in groups 4 and 5, and significantly lower in group 5 than those in group 4. Additionally, the protein expressions of vascular endothelial growth factor (VEGF), C-X-C motif chemokine receptor 4 (CXCR4), and stromal cell-derived factor (SDF)-1α, other three indicators of angiogenesis, were progressively and significantly increased from groups 1 to 3, 5 and 4, suggesting an intrinsic response to ischemic stimulation that was further enhanced by ECSW treatment.

**Figure 5:**
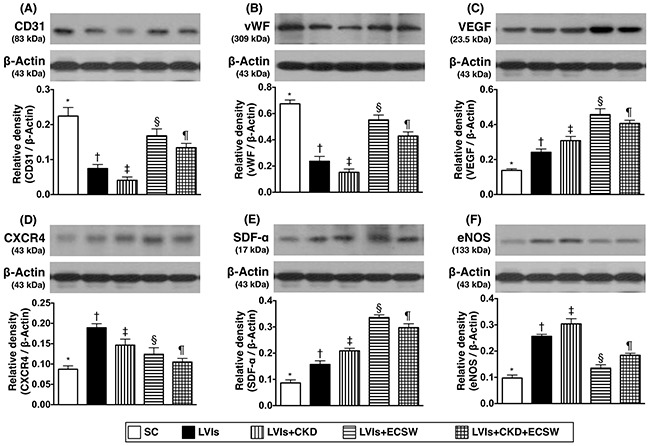
Protein expressions of angiogenesis factors in LV myocardium by day 180 after CKD induction **(A)** Protein expression of CD31, * vs. other groups with different symbols (†, ‡, §, ¶), p<0.0001. **(B)** Protein expression of von Willebrand factor (vWF), * vs. other groups with different symbols (†, ‡, §, ¶), p<0.0001. **(C)** Protein expression of vascular endothelial growth factor (VEGF), * vs. other groups with different symbols (†, ‡, §, ¶), p<0.0001. **(D)** Protein expression of CXCR4, * vs. other groups with different symbols (†, ‡, §, ¶), p<0.001. **(E)** Protein expression of stromal cell-derived factor (SDF)-α, * vs. other groups with different symbols (†, ‡, §, ¶), p<0.0001. **(F)** Protein expression of endothelial nitric oxide synthase (eNOS), * vs. other groups with different symbols (†, ‡, §, ¶), p<0.0001. All statistical analyses were performed by one-way ANOVA, followed by Bonferroni multiple comparison post hoc test (n=6 for each group). Symbols (*, †, ‡, §, ¶) indicate significance (at 0.05 level). SC: sham-operative control; LVIs: left ventricular ischemia; CKD: chronic kidney disease; ECSW: extracorporeal shock wave.

### Protein expressions of oxidative stress biomarkers in LV myocardium by day 180 after CKD induction (Figure 6)

The protein expressions of NADPH oxidase (NOX)-1, NOX-2, and oxidized protein, three indices of oxidative stress, were lowest in group 1 and highest in group 3, significantly higher in group 2 than those in groups 4 and 5, and significantly higher in group 5 than those in group 4.

**Figure 6:**
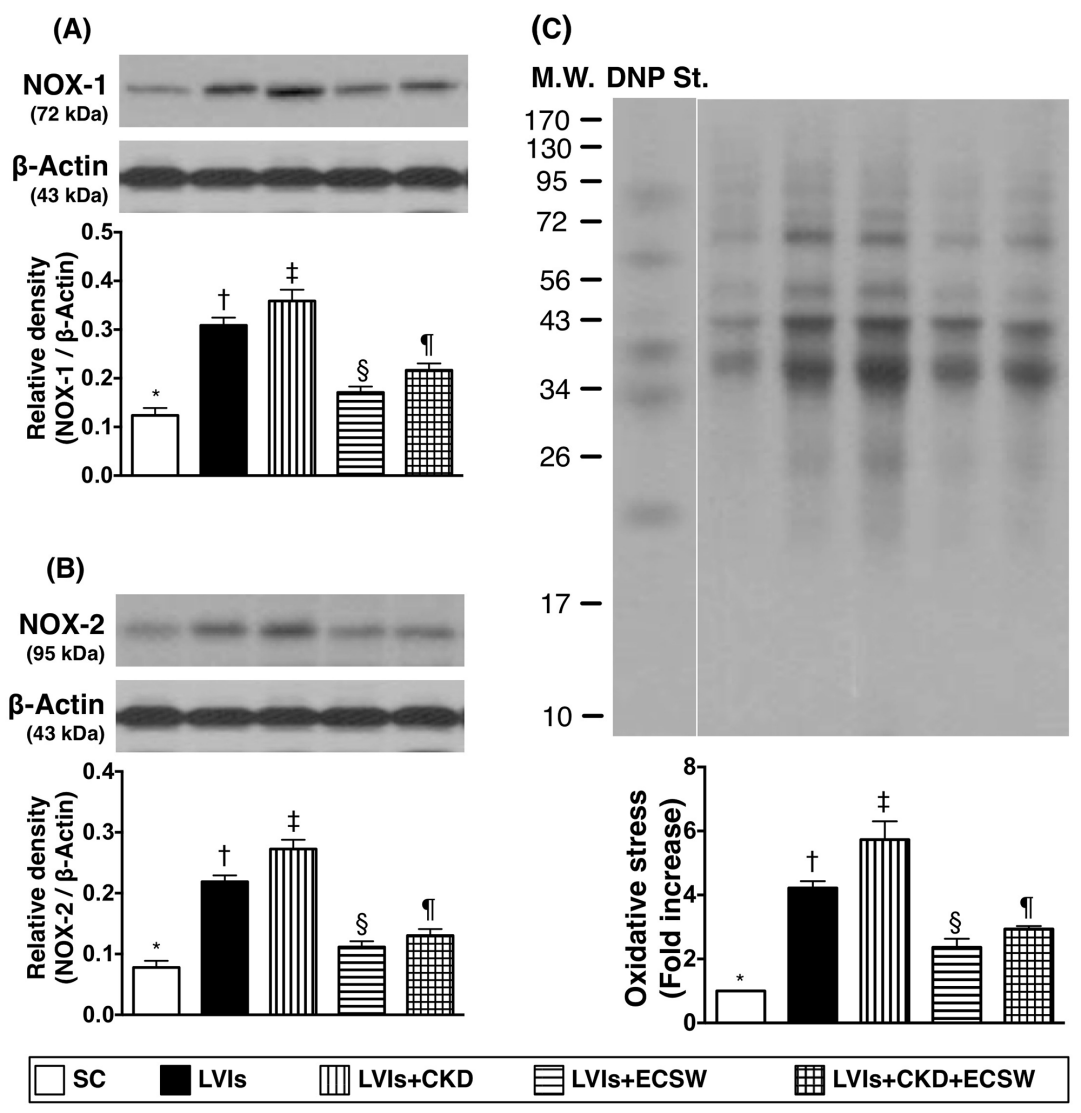
Protein expressions of oxidative stress biomarkers in LV myocardium by day 180 after CKD induction **(A)** Protein expression of ONX-1, * vs. other groups with different symbols (†, ‡, §, ¶), p<0.0001. **(B)** Protein expression of ONX-2, * vs. other groups with different symbols (†, ‡, §, ¶), p<0.0001. **(C)** Oxidized protein expression, * vs. other groups with different symbols (†, ‡, §, ¶), p<0.0001. (Note: left and right lanes shown on the upper panel represent protein molecular weight marker and control oxidized molecular protein standard, respectively). M.W: molecular weight; DNP: 1-3 dinitrophenylhydrazone. All statistical analyses were performed by one-way ANOVA, followed by Bonferroni multiple comparison post hoc test (n=6 for each group). Symbols (*, †, ‡, §, ¶) indicate significance (at 0.05 level). SC: sham-operative control; LVIs: left ventricular ischemia; CKD: chronic kidney disease; ECSW: extracorporeal shock wave.

### Protein expressions of mitochondrial integrity, pressure/volume overload and myocardial hypertrophy biomarkers in LV myocardium by day 180 after CKD induction (Figure 7)

The protein expression of peroxisome proliferator activated receptor-gamma coactivator 1 alpha (PGC-1α), a transcriptional coactivator and a major upstream regulator of lipid catabolism, oxidative metabolism, mitochondrial metabolism, and biogenesis [34, 35], was lowest in group 3 and highest in group 1, significantly lower in group 2 than that in groups 4 and 5, and significantly lower in group 5 than that in group 4. Moreover, the protein expression of mitochondrial cytochrome C, an indicator of mitochondrial integrity, showed a pattern identical to that of PGC-α among the five groups. On the other hand, the protein expression of cytosolic cytochrome C, an indicator of mitochondrial damage, displayed an opposite pattern compared to that of PGC-α.

**Figure 7:**
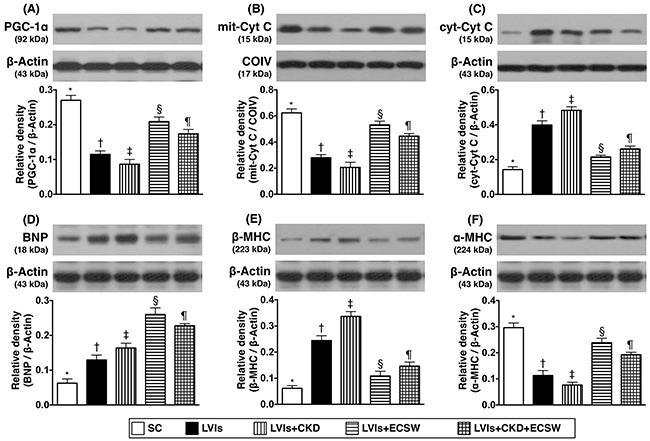
Protein expressions of mitochondrial integrity, pressure/volume overload and myocardial hypertrophy biomarkers in LV myocardium by day 180 after CKD induction **(A)** Protein expression of peroxisome proliferator activated receptor-gamma coactivator 1 alpha (PGC-α), * vs. other groups with different symbols (†, ‡, §, ¶), p<0.0001. **(B)** Protein expression of mitochondrial cytochrome C (mit-Cyt C), * vs. other groups with different symbols (†, ‡, §, ¶), p<0.0001. **(C)** Protein expression of cytosolic cytochrome C (cyt-Cyt C), * vs. other groups with different symbols (†, ‡, §, ¶), p<0.0001. **(D)** Protein expression of brain natriuretic peptide (BNP), * vs. other groups with different symbols (†, ‡, §, ¶), p<0.0001. **(E)** Protein expression of beta myosin heavy chain (β-MHC), * vs. other groups with different symbols (†, ‡, §, ¶), p<0.0001. **(F)** Protein expression of α-MHC, * vs. other groups with different symbols (†, ‡, §, ¶), p<0.0001. All statistical analyses were performed by one-way ANOVA, followed by Bonferroni multiple comparison post hoc test (n=6 for each group). Symbols (*, †, ‡, §, ¶) indicate significance (at 0.05 level). SC: sham-operative control; LVIs: left ventricular ischemia; CKD: chronic kidney disease; ECSW: extracorporeal shock wave.

Furthermore, the protein expressions of brain natriuretic peptide (BNP) and β- myosin heavy chain (MHC) [36], two pressure overload/heart failure biomarkers, showed an opposite pattern compared to that of PGC-α among the five groups. Conversely, protein expression of α-MHC, a reverse myocardial hypertrophic biomarker, showed a pattern opposite to that of β-MHC among the five groups.

### Protein expressions of fibrosis and apoptotic biomarkers in LV myocardium by day 180 after CKD induction (Figure 8)

The protein expressions of transforming growth factor (TGF)-β and Smad3, two indicators of fibrosis, were lowest in group 1 and highest in group 3, significantly higher in group 2 than those in groups 4 and 5, and significantly higher in group 5 than those in group 4. On the other hand, the protein expressions of bone morphogenetic protein 2 (BMP2) and Smad1/5, two anti-fibrotic biomarkers, showed an opposite pattern compared to that of TFG-β among the five groups.

**Figure 8:**
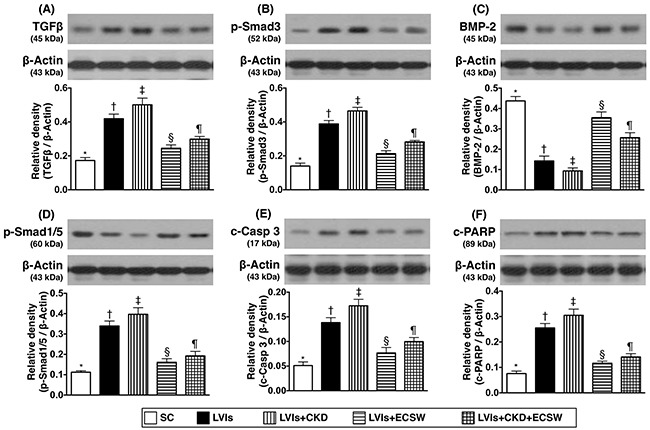
Protein expressions of fibrosis and apoptotic biomarkers in LV myocardium by day 180 after CKD induction **(A)** Protein expression of transforming growth factor (TGF)-β, * vs. other groups with different symbols (†, ‡, §, ¶), p<0.0001. **(B)** Protein expression of phosphorylated (p)-Smad3, * vs. other groups with different symbols (†, ‡, §, ¶), p<0.0001. **(C)** Protein expression of bone morphogenetic protein (BMP)-2, * vs. other groups with different symbols (†, ‡, §, ¶), p<0.0001. **(D)** Protein expression of p-Smad1/5, * vs. other groups with different symbols (†, ‡, §, ¶), p<0.0001. **(E)** Protein expression of cleaved (c)-caspase 3, * vs. other groups with different symbols (†, ‡, §, ¶), p<0.0001. **(F)** Protein expression of cleaved poly (ADP-ribose) polymerase (c-PARP), * vs. other groups with different symbols (†, ‡, §, ¶), p<0.0001. * vs. other groups with different symbols (†, ‡, §, ¶), p<0.0001. All statistical analyses were performed by one-way ANOVA, followed by Bonferroni multiple comparison post hoc test (n=6 for each group). Symbols (*, †, ‡, §, ¶) indicate significance (at 0.05 level). SC: sham-operative control; LVIs: left ventricular ischemia; CKD: chronic kidney disease; ECSW: extracorporeal shock wave.

In addition, the protein expressions of cleaved caspase 3 and cleaved poly(ADP-ribose) polymerase (PARP), two indicators of apoptosis, exhibited a pattern identical of that of TFG-β among the five groups.

## DISCUSSION

This study, which investigated the therapeutic effect of ECSW treatment against CKD- and ischemia-related LV dysfunction and LV remodeling in the setting of CRS, yielded several important clinical implications. First, CKD significantly contributed to ischemia-related LV dysfunction and remodeling. Second, ECSW treatment markedly preserved CRS-induced LV dysfunction and remodeling, highlighting the therapeutic potential of ECSW in the treatment of patients with diffuse CAD in the clinical setting of CKD/ESRD as well as those refractory to medical treatment and also unsuitable for coronary interventions. Third, angiogenesis is the major mechanism by which LVEF was improved.

CKD is frequently associated with CAD and acute coronary syndrome [10, 12, 37, 38]. Although it is well recognized that there are various etiologies for coronary arterial calcification and diffuse CAD, CDK/ESRD is undoubtedly one of the most important contributes [10, 12, 37–40]. An essential finding in the present study is that ischemia-induced LV dysfunction and remodeling are aggravated in the CKD setting. This finding implies that the adverse effects of CKD on LV function not only include the induction of coronary artery calcification and diffuse obstructive CAD [10, 12, 37–40], but may also involve other pathways.

The associations among inflammation, oxidative stress, fibrosis, cellular apoptosis, and LV dysfunction have been shown in the experimental model of doxorubicin-induced dilated cardiomyopathy [36], ischemic heart disease [15, 41–43], and CRS [11]. Intriguingly, when we looked at the expressions of these biomarkers at molecular-cellular levels in LV myocardium, we found that the fibrosis/collagen-deposition areas, numbers of apoptotic nuclei, and levels of oxidative stress were substantially increased in animals with LVIs and further substantially increased in animals with LVIs + CKD (i.e., CRS). These findings, in addition to reinforcing those of previous studies [11], highlight that the impact of CKD on LV function and remodeling may be multi-factorial, including anatomical distortions and molecular-cellular perturbations.

Universal consensus is that calcified/diffuse CAD caused by ESRD remains a formidable challenge for intervention cardiologists [1, 3, 4] because an effective tool for management is currently unavailable [2, 17]. A principal finding in the present study is that, as compared with the SC animals, LVEF was remarkably reduced in animals with LVIs and further remarkably reduced in the LVIs-CKD group, implicating a role of CKD in aggravating LV dysfunction in an ischemic heart setting (i.e., ameroid constrictor in LAD). The most important finding in the present study is that, as compared with LVIs/LVIs-CKD animals, LVEF was significantly preserved in LVIs-CKD animals and further significantly preserved in LVIs animals after receiving ECSW treatment. This finding, in addition to explaining the notably suppressed expressions of pressure/volume overload, LV hypertrophy and remodeling biomarkers (i.e., BNP, β-BMC) in LVIs/LVIs-CKD + ECSW animals, raises the need for a clinical prospective study to evaluate the effectiveness of ECSW treatment in patients with CKD/ESRD who have calcified/diffuse CAD unsuitable for coronary interventions (i.e., PCI or CABG) and refractory to optimal medication for angina pectoris.

Experimental studies have demonstrated that angiogenesis/neovascularization in ischemic tissue effectively restores blood flow and improved ischemia-related organ dysfunction [15, 16, 18–20, 41, 42]. Additionally, previous clinical trials have shown that EPC therapy enhanced angiogenesis and improved angina and LV dysfunction in patients with diffuse CAD [2, 17]. Furthermore, a limited number of clinical studies [30–32] and abundant experimental investigations [15, 19, 42] have shown that ECSW could augment angiogenesis in ischemic tissue/organ. One important finding in the present study is that not only were molecular perturbations (i.e., fibrosis, apoptosis, oxidative stress, and volume/pressure overload biomarkers) markedly suppressed, but pro-angiogenic factors were also upregulated in LVIs/LVIs-CKD animals after ECSW treatment. Our findings, in addition to strengthening those from previous studies [15, 19, 30–32, 42], could at least in part explain the notable improvement in LVEF and the significant suppression of LV remodeling in LVIs/LVIs-CKD animals compared to those animals with LVIs without ECSW treatment.

Our previous studies have shown that LV dysfunction was strongly associated with energy deprivation [15, 36, 42]. Another important finding of the present study is that the protein expression of cytosolic cytochrome C (i.e., mitochondrial damage) was remarkably increased and the protein expressions of mitochondrial cytochrome C and PGC-1α (i.e., mitochondrial integrity) substantially dropped in LVIs/LVIs-CKD animals compared to those in SC animals. However, the expressions of these three parameters were notably reversed in LVIs/LVIs-CKD animals after receiving ECSW treatment. These findings, in addition to corroborating those of our previous studies [15, 36, 42], could once again explain the significant preservation of LVEF in LVIs/LVIs-CKD animals following ECSW treatment.

### Study limitation

This study has limitations. First, although extensive works had been done in the current study, the exact underlying mechanisms had not been completely investigated. Second, other two groups, i.e., (1) SC + ECSW therapy and (2) CKD only were not included in the present study. This is out of the scope of this study because the original protocol was not designed to include the two groups in order to reduce the use of animals according to principle of replacement, reduction, and refinement. Third, this experimental model is really not identical to the patients with clinical setting of diffuse CAD. Finally, the animal sample size of each group was relative small. Therefore, we cannot completely rule out the statistical significance had been distorted in the present study.

In conclusion, the results of the current study demonstrated that CKD is a significant contributor to ischemia-related LV dysfunction and remodeling that were abrogated by ECSW treatment.

## MATERIALS AND METHODS

### Ethics

Both animal experimental protocols and procedures were approved by our Institute of Animal Care and Use Committee at Kaohsiung Chang Gung Memorial Hospital (Affidavit of Approval of Animal Use Protocol No. 2014121813) and executed in accordance with the Guide for the Care and Use of Laboratory Animals [The Eighth Edition of the Guide for the Care and Use of Laboratory Animals (NRC 2011)].

Animals were housed in an Association for Assessment and Accreditation of Laboratory Animal Care International (AAALAC)-approved animal facility in our hospital with controlled temperature and light cycle (24°C and 12/12 light-dark cycle).

### Creation of chronic kidney disease and ischemic heart disease models and experimental protocol

The establishment of an animal model of chronic myocardial ischemia was based on our previous report [15]. In details, male mini-pig (Taitung Animal Propagation Station, Livestock Research Institute, Taiwan), weighting 16-18 kg, was anesthetized by intramuscular injections of ketamine (15 mg/kg) and maintained with an inhalation of 1.5% isoflurane during the procedure. After being shaved on the chest and abdomen, the mini-pig was placed in supine position on a warming pad at 37 °C followed by endotracheal intubation with positive-pressure ventilation (180 mL/min) with room air using a ventilator (Sn: Q422ZO, SIMS PneuPAC, Ltd.). Electrocardiographic (ECG) monitor and defibrillator were then connected to the chest wall.

Under sterile conditions, the heart was exposed through mid-thoracotomy. After gentle removal of the pericardium, an ameroid constrictor (Research Instruments NW, Inc. Lebanon, OR, USA) was applied to the mid-left anterior descending artery (LAD) just beyond the first diagonal branch. The constrictor constitutes an outer layer of stainless steel and an inner layer of fluid-absorbing casein that swells as it slowly absorbs body fluid to produce an inward concentric expansion. It usually required several weeks to create a significant stenosis over the mid-portion of LAD.

Neither regional ischemia nor wall motion abnormality was noted over LV anterior wall after the procedure. The absence of cardiac ischemia was further confirmed by complete ECG following the procedure. The chest wound was then closed in layers.

Chronic kidney disease (CKD) was induced in mini-pigs two weeks prior to the performance of chronic myocardial ischemia procedure. The animals were anesthetized by inhalational 2.0% isoflurane and placed in a supine position on a warming pad at 37 °C for midline laparotomies. Right nephrectomy was performed and half (1/2) of the left renal arterioles were ligated. The abdominal wall was closed in layers. The animals were allowed to remain on the warming pad and recover under care.

Thirty mini-pigs were equally and randomly divided into group 1 [sham-control (SC)], group 2 [LV-ischemia (LVIs)], group 3 (LVIs + CKD), group 4 [LVIs + ECSW [applied 1200 shots at 0.1 mJ/m^2^/equally to 4 ischemic regions (i.e., each ischemic region received totally 300 shots) by day-90 after LVIs procedure], and group 5 (LVIs + CKD + ECSW).

### Procedure and protocol for application of ECSW to LV myocardium of mini-pig

The procedure and protocol of applying ECSW to LV ischemic zones were based on our previous report [15, 42] with minimal modification. In details, by day 90 after induction of chronic heart ischemia, each mini-pig in groups 4 and 5 was anesthetized again without endotracheal intubation. ECSW treatment was given to groups 4 and 5 animals using a shock wave generating system (Cardiospec, Medispec, Israel). An echocardiographic imaging system was utilized to locate the treatment areas of left ventricle. Under transthoracic sonographic guidance, shock waves were then delivered through a special applicator via anatomical acoustic windows to the treatment areas under ECG R-wave gating. Briefly, after echocardiographic localization of the four ischemic zones of LV (i.e., middle anterior, middle anteroseptal, middle inferior, and middle inferolateral segments) using parasternal axis approach, ECSW was applied once to each of the four ischemic zones at an intensity of 300 impulses at 0.1 mJ/mm^2^/zone so that the total energy applied to LV myocardium was 1200 impulses at 0.1 mJ/mm^2^.

### Functional assessment by transthoracic echocardiography

Transthoracic echocardiography was performed preoperatively and on days 90 and 180 after the procedure under general anesthesia as described previously [42, 43] using iE33 (Philips Medical System, Bothell, WA) with X5-1 transducers. In details, end-diastolic dimension (EDD) and end-systolic dimension (ESD) were assessed at mitral valve level and papillary level of left ventricle. Recordings were stored for off-line two-dimensional image analysis using a computer software (Q-lab v6; Philips Medical System). For each mini-pig, three consecutive beats were measured and averaged for each variable. With the animals in a supine position, left ventricular internal dimensions [i.e. end-systolic diameter (ESD) and end-diastolic diameter (EDD)] were examined according to the American Society of Echocardiography leading-edge method using at least 3 consecutives cardiac cycles. The LV ejection fraction (LVEF) was calculated as: LVEF (%) = [(LVEDD^3^-LVEDS^3^)/LVEDD^3^] x 100. Intra-observer variability was evaluated in a series of 6 mini-pigs by one observer who examined each mini-pig recording in a consecutive way. Intra-observer variability for LVEF was 2.7 ± 2.2%. All measurements were performed by a cardiologist blinded to the treatment and non-treatment groups.

### Assessment of circulating levels of creatinine and flow cytometric analysis for determining the circulating level of endothelial progenitor cells (EPCs)

Blood samplings at 8:00 a.m. as baseline and at days 3, 30, 90 and 180 after CKD were performed to determine the circulating level of creatinine by standard laboratory method. Additionally, by day 180 prior to euthanize each group of animals, blood was also sampled at 8:00 a.m. for determining the circulating level of EPCs (i.e., CD31^+^CD117^+^, CD34^+^CD117^+^) by flow cytometry based on the method of our previous reports [41, 42].

### Kidney injury scores at day 180 after CKD induction

Histopathology scoring was assessed in a blinded fashion as we have previously described [44]. Briefly, the kidney specimens from all animals were fixed in 10% buffered formalin, embedded in paraffin, sectioned at 5 μm and stained with hematoxylin and eosin (H & E)for light microscopy. The scoring system reflects the grading of tubular necrosis, loss of brush border, cast formation, and tubular dilatation in 10 randomly chosen, non-overlapping fields (200x) as follows: 0 (none), 1 (≤10%), 2 (11–25%), 3 (26–45%), 4 (46–75%), and 5 (≥76%).

### Western blot analysis

The procedure was based on our recent report [11, 41–44]. In details, equal amounts (50μg) of protein extracts were loaded and separated by SDS-PAGE using 8-12% acrylamide gradients. After electrophoresis, the separated proteins were transferred electrophoretically to a polyvinylidene difluoride (PVDF) membrane (Amersham Biosciences). Nonspecific sites were blocked by incubation of the membrane in blocking buffer [5% nonfat dry milk in T-TBS (TBS containing 0.05% Tween 20)] overnight. The membranes were incubated with the indicated primary antibodies [cleaved caspase 3 (1: 1000, Cell Signaling), cleaved poly (ADP-ribose) polymerase (PARP) (1: 1000, Cell Signaling), phosphorylated (p)-Smad3 (1: 1000, Cell Signaling), p-Smad1/5 (1: 1000, Cell Signaling), bone morphogenetic protein (BMP)-2 (1:500, Abcam), transforming growth factor (TGF)-β (1:500, Abcam), CD31 (1:1000, Abcam), von Willebrand factor (vWF) (1:1000, Abcam), CXCR4 (1:1000, Abcam), vascular endothelial growth factor (VEGF) (1:1000, Abcam), stromal cell-derived factor (SDF)-1α (1: 1000, Cell Signaling), α-smooth muscle actin (SMA) (1: 1000, Sigma), peroxisome proliferator activated receptor-gamma coactivator 1 alpha (PGC-1α) (1:1000, Abcam), cytosolic cytochrome C (1:1000, Millipore), mitochondrial cytochrome C (1:1000, Millipore), α-myosin heavy chain (MHC) (1:500, Santa Cruz), β-MHC (1:750, Santa Cruz), NOX-1 (1: 1500, Sigma), NOX-2 (1:750, Sigma) and α-brain natriuretic peptide (BNP) (1: 1000, Cell Signaling)] for 1 hour at room temperature. Horseradish peroxidase-conjugated anti-rabbit immunoglobulin IgG (1:2000, Cell Signaling) was used as the secondary antibody for one-hour incubation at room temperature. The washing procedure was repeated eight times within an hour, and immunoreactive bands were visualized by enhanced chemiluminescence (ECL; Amersham Biosciences) after exposure to Biomax L film (Kodak). For purposes of quantification, ECL signals were digitized using Labwork software (UVP).

### Assessment of oxidative stress reaction

The Oxyblot Oxidized Protein Detection Kit was purchased from Chemicon (S7150). The procedure was according to our recent study [11, 42, 45]. DNPH derivatization was carried out on 6 μg of protein for 15 minutes according to manufacturer's instructions. One-dimensional electrophoresis was carried out on 12% SDS/polyacrylamide gel after DNPH derivatization. Proteins were transferred to nitrocellulose membranes, which were then incubated in the primary antibody solution (anti-DNP 1:150) for 2 hours, followed by incubation with secondary antibody solution (1:300) for one hour at room temperature. The washing procedure was repeated eight times within 40 minutes. Immunoreactive bands were visualized by enhanced chemiluminescence (ECL), which was then exposed to Biomax L film (Fuji). For quantification, ECL signals were digitized using Labwork software (UVP). On each gel, a standard control sample was loaded.

### Immunofluorescent (IF) staining

The procedure and protocol of IF staining have been reported in details in our previous and recent reports [41–45]. For IF staining, rehydrated paraffin sections were first treated with 3% H_2_O_2_ for 30 minutes and incubated with Immuno-Block reagent (BioSB, Santa Barbara, CA, USA) for 30 minutes at room temperature. Sections were then incubated with primary antibodies specifically against CD31 (1:100, BIO-RAD, CA, USA), vWF (1:200, Millipore, Massachusetts, USA) and TUNEL assay for apoptotic nuclei (In Situ Cell Death Detection Kit POD, Roche, Basel, Switzerland), while sections incubated with the use of irrelevant antibodies served as controls. Three sections of LV specimen from each rat were analyzed. For quantification, three randomly selected HPFs (400x for IF study) were analyzed in each section. The mean number of positively-stained cells per HPF for each animal was then determined by summation of all numbers divided by 9.

### Histological study of fibrosis and collagen-deposition area

Masson's trichrome and Sirius red staining were used for studying fibrosis and collagen deposition in LV myocardium. Three 4 μm thick serial sections of LV myocardium were prepared by Cryostat (Leica CM3050S). The integrated area (μm^2^) of fibrosis in each section was calculated using Image Tool 3 (IT3) image analysis software (University of Texas, Health Science Center, San Antonio, UTHSCSA; Image Tool for Windows, Version 3.0, USA). Three selected sections were quantified for each animal. Three randomly selected HPFs (100x) were analyzed in each section. After determining the number of pixels in each fibrotic area per HPF, the numbers of pixels obtained from the three HPFs were summated. The procedure was repeated in two other sections for each animal. The mean pixel number per HPF for each animal was then determined by summating all pixel numbers and dividing by 9. The mean integrated area (μm^2^) of fibrosis in LV myocardium per HPF was obtained using a conversion factor of 19.24 (1 μm^2^ represented 19.24 pixels).

### Vessel density in LV myocardium

Immunohistochemical staining of blood vessels was performed with alpha-smooth actin (α-SMA) (1:400) as primary antibody at room temperature for one hour, followed by washing with PBS thrice. The anti-mouse-HRP conjugated secondary antibody was then added for 10 minutes, followed by washing with PBS thrice. Then 3,3’ diaminobenzidine (DAB) (0.7 gm/tablet) (Sigma) was added for one minute, followed by washing with PBS thrice. Finally, hematoxylin was added for one minute as a counter-stain for nuclei, followed by washing twice. For quantification, three sections of infarct area were chosen for each rat and three randomly selected high-power field (HPF) (x100) were analyzed for each section. The mean number per HPF for each animal was then determined by summation of all numbers divided by 9.

### Statistical analysis

Quantitative data are expressed as means ± SD. Statistical analysis was adequately performed by ANOVA followed by Bonferroni multiple-comparison post hoc test. Statistical analysis was performed using SAS statistical software for Windows version 8.2 (SAS institute, Cary, NC). A probability value <0.05 was considered statistically significant.
